# Large-Area Fluorescence and Electron Microscopic Correlative Imaging With Multibeam Scanning Electron Microscopy

**DOI:** 10.3389/fncir.2019.00029

**Published:** 2019-05-08

**Authors:** Shinsuke Shibata, Taro Iseda, Takayuki Mitsuhashi, Atsushi Oka, Tomoko Shindo, Nobuko Moritoki, Toshihiro Nagai, Shinya Otsubo, Takashi Inoue, Erika Sasaki, Chihiro Akazawa, Takao Takahashi, Richard Schalek, Jeff W. Lichtman, Hideyuki Okano

**Affiliations:** ^1^Electron Microscope Laboratory, Keio University School of Medicine, Tokyo, Japan; ^2^Department of Physiology, Keio University School of Medicine, Tokyo, Japan; ^3^Department of Pediatrics, Keio University School of Medicine, Tokyo, Japan; ^4^Central Institute for Experimental Animals, Kawasaki, Japan; ^5^Department of Biochemistry and Biophysics, Graduate School of Health Care Sciences, Tokyo Medical and Dental University, Tokyo, Japan; ^6^Department of Molecular and Cellular Biology, Harvard University, Cambridge, MA, United States; ^7^Laboratory for Marmoset Neural Architecture, RIKEN Center for Brain Science, Wakō, Japan

**Keywords:** correlative imaging, immuno-EM, CLEM, connectomics, multibeam SEM

## Abstract

Recent improvements in correlative light and electron microscopy (CLEM) technology have led to dramatic improvements in the ability to observe tissues and cells. Fluorescence labeling has been used to visualize the localization of molecules of interest through immunostaining or genetic modification strategies for the identification of the molecular signatures of biological specimens. Newer technologies such as tissue clearing have expanded the field of observation available for fluorescence labeling; however, the area of correlative observation available for electron microscopy (EM) remains restricted. In this study, we developed a large-area CLEM imaging procedure to show specific molecular localization in large-scale EM sections of mouse and marmoset brain. Target molecules were labeled with antibodies and sequentially visualized in cryostat sections using fluorescence and gold particles. Fluorescence images were obtained by light microscopy immediately after antibody staining. Immunostained sections were postfixed for EM, and silver-enhanced sections were dehydrated in a graded ethanol series and embedded in resin. Ultrathin sections for EM were prepared from fully polymerized resin blocks, collected on silicon wafers, and observed by multibeam scanning electron microscopy (SEM). Multibeam SEM has made rapid, large-area observation at high resolution possible, paving the way for the analysis of detailed structures using the CLEM approach. Here, we describe detailed methods for large-area CLEM in various tissues of both rodents and primates.

## Introduction

Comprehensive investigation of neural circuits in relatively large and complex brains such as those of humans and marmosets requires simultaneous low- and high-magnification observations within each layer of the cerebral cortex. To elucidate the structural interconnection between neurons in neocortices at both of these levels, connectomics analysis based on the knowledge of neocortical layer development is critical. Neocortical development involves three key processes: neurogenesis, migration and differentiation/maturation. The mature mammalian neocortex has a six-layered structure; the neurons in each layer of the neocortex are generated by division of neural stem/progenitor cells that surround the lateral ventricles of the embryonic forebrain (Sidman et al., 1959; Takahashi et al., 1995). These neurons migrate radially toward the pial surface in an inside-out manner (Rakic, 1972) and express a specific pattern of “marker” proteins (Hevner, 2007). Thus, by detecting these layer-specific markers using immunohistochemical staining or *in situ* hybridization (ISH), it is theoretically possible to specify the layer position of specific neurons of interest in which the synapses have been analyzed by EM. However, such analyses have been constrained by technical challenges due to the fact that procedures that combine results from light microscopy (LM) and EM require the use of different instruments and sample preparation methods and by the fact that both LM and EM demand high levels of expertise. CLEM has begun to enable the elucidation of subcellular architectures and morphologies (Begemann and Galic, 2016). Traditionally, CLEM is performed by correlating results obtained from LM and TEM. Fluorescence microscopy has the advantage of visualizing immunolabels that recognize specific molecules using antibodies or fluorescent proteins such as GFP (Giepmans, 2008; Watanabe et al., 2011). Fluorescent dyes can be distributed to a target area or to molecules in a relatively wider field with optimal efficiency and can be detected by LM. However, the spatial resolution of conventional LM is restricted to a few hundred nanometers at best due to the diffraction of light. Super-resolution light microscopy was developed to overcome this diffraction barrier, and its developers were recognized with the Nobel Prize in Chemistry in 2014 (Chereau et al., 2015). Because fluorescence imaging inherently focuses on labeled objects, peripheral cellular structures often remain poorly visualized. EM yields much higher-resolution images than LM but is difficult to use to observe large tissue areas or to make precise observations of highly dynamic processes such as those that occur in the human brain or in living cells (Giepmans, 2008; Watanabe et al., 2011; Chereau et al., 2015). Although CLEM has been used for decades, until recently it has only been applied to small-volume samples. The development of improved CLEM techniques has enabled scientists to achieve nanometer resolution analyses in samples that are more than several mm^2^ in area, including samples of the gyrencephalic brain (Eberle et al., 2015a). Using multibeam SEM, we have developed a novel implementation, LA-CLEM, that offers additional advantages for the detection of molecular localization in large areas of the CNS at EM resolution and faster speeds. Visual information provided by layer-specific markers in EM images proved helpful in understanding the precise location of observed samples, particularly in the cerebrum of the common marmoset, which is much larger than that of mouse.

**Figure 1:**
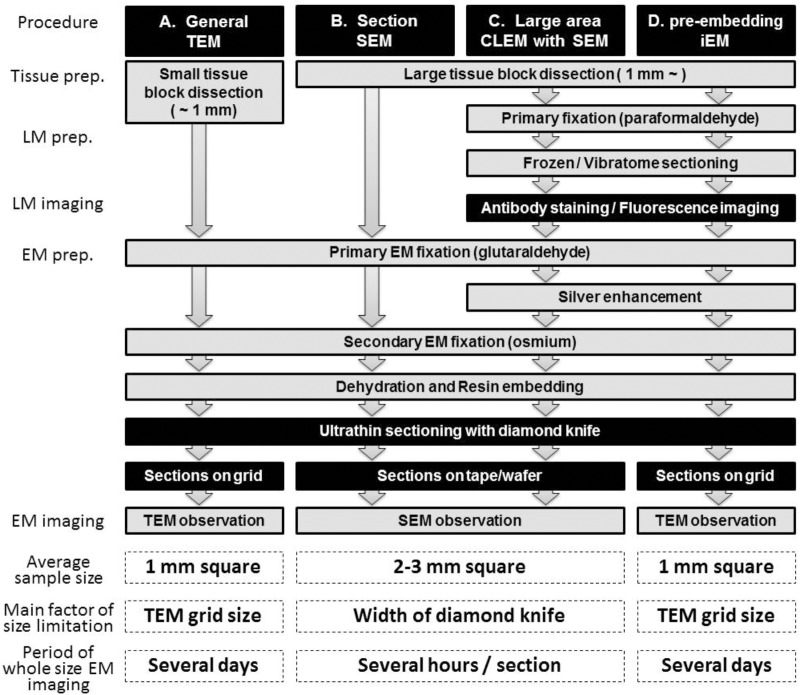
Schematic illustration of LA-CLEM. Sample preparation procedures for general TEM **(A)**, section SEM **(B)**, large-area CLEM (LA-CLEM) with SEM **(C)** and pre-embedding immune-EM **(D)** are shown. Human biopsy or autopsy specimens, marmoset brain specimens and rodent tissue samples have typically been processed for general TEM observation and for diagnostic purposes. More recently, large-area observation of tissue sections has been conducted using SEM, including multibeam SEM. Localization of proteins and nucleotides (RNA/DNA) is possible using iEM. The combination of large-area observation with iEM is the key to LA-CLEM imaging. Detailed procedures for LA-CLEM are presented in the main text. Numerical comparisons of the average sample size, the main factor of size limitation, and the period of whole size EM imaging between each procedure are provided at the bottom of this chart. The average sample size for TEM observation is a section approximately 1 mm^2^ **(A,D)**. It requires several days to image the entire 1 mm^2^ area on the grid. In contrast, a 2–3 mm^2^ section is the average size restricted by the width of the diamond knife, and it requires several hours to image the entire 2–3 mm^2^ area on the silicon wafer.

Transmission electron microscopy of ultrathin sections obtained from human biopsy or autopsy samples or rodent brain and collected on an EM grid has traditionally been used to observe synaptic connections between neurons (Figure 1A). In this process, brain tissues are dissected into small pieces of <1 mm and fixed with glutaraldehyde and osmium. The brain tissue block embedded in the plastic is sectioned at a thickness of approximately 50–80 nm using a diamond knife, and the sections are collected on an EM grid. This procedure remains in common use for the observation of synaptic structure. Recent improvements in the resolution of SEM images now enable the observation of synaptic structure by back-scattered electron imaging and by secondary electron imaging. For large-area EM observations, section SEM is now frequently used (Figure 1B). In this procedure, sample preparation is similar to that for TEM except for the collection of the ultrathin sections on flat conductive substances including silicon wafers, conductive coated glass, or conductive tape rather than on an EM grid. Observation of neural circuitry by EM, when combined with visualization of specific layer components in the cerebrum by fluorescence and EM, yields an unprecedented depth of information on the complex features of the gyrencephalic brain. Below, we introduce a new approach, LA-CLEM, that makes it possible to observe samples several millimeters square in area at resolutions that make it possible to detect individual synapses (Figure 1C). To identify the cerebral layer in which these synapses reside, the most common approach is the use of antibody staining or ISH to label layer-specific markers. The localization of specific target proteins and nucleotides (RNA/DNA) was demonstrated not only by fluorescence at the LM level but also by gold with iEM (immuno-EM) at the EM level (Figure 1D). Antibodies against layer-specific marker molecules, including antibodies against calbindin, calretinin, RORβ, Cux1, and FoxP2, are often used to evaluate normal layer formation. By combining immuno-EM and large-area SEM imaging, LA-CLEM can be used to visualize the localization of specific molecules in large areas at super-high resolution.

In this report, we present the detailed procedure of LA-CLEM, a combination approach involving pre-embedded immuno-EM and multibeam SEM technology that has been adapted for use in the marmoset cerebral cortex.

## Sample Preparation

### Animals

Adult common marmoset monkeys (*Callithrix jacchus*, CLEA, Tokyo, Japan, *n* = 3), adult mice (*Mus musculus*, C57BL6/j from Japan SLC, Shizuoka, Japan, *n* = 10), and Sox10-Venus BAC transgenic mice (*n* = 4) (Shibata et al., 2010) were used in this study. Housing of animals and all animal experiments were conducted in compliance with the Guidelines for the Care and Use of Laboratory Animals of Keio University School of Medicine (approval numbers 11006-2 and 09091-12) and the Central Institute for Experimental Animals (approval numbers 16023 and 17031). All efforts were made to reduce the number of animals used and to minimize animal suffering.

### PRIMARY SAMPLE PREPARATION FOR LA-CLEM

**Figure 2:**
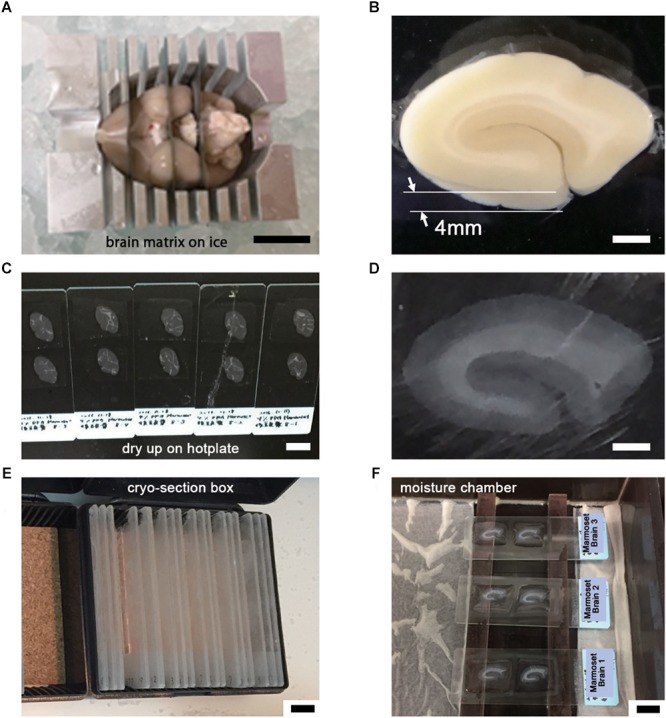
Tissue preparation for LA-CLEM observation. **(A)** After perfusion with 4% PFA pH 7.4 in PBS, brain tissue was dissected into coronal blocks 3–6 mm in thickness suitable for cryomolds (Tissue-Tek) with the brain matrix on ice. **(B)** The target brain area was dissected under an optical microscope using a blade. In this experiment, a whole coronal block from the occipital lobe of marmoset brain was prepared at 4 mm thickness. **(C–E)** Frozen sections at 20 μm thickness were prepared using a cryostat; completely dried sections on slides were stored in a cryosection box and preserved in a freezer at –30 or –80°C. **(F)** The sections were thawed and redried, followed by the application of blocking solution on the day of antibody staining. Scale bars: **(A)** 1 cm, **(B)** 2 mm, **(C)** 1 cm, **(D)** 2 mm, **(E)** – **(F)** 1 cm.

The basic procedure used in immunohistochemical analysis was performed as described previously (Shibata et al., 2010). Briefly, animals were deeply anesthetized by intramuscular injection of ketamine (50 mg/kg, Fujita Pharmaceutical, Tokyo, Japan) and xylazine (4 mg/kg, Bayer, Leverkusen, Germany) for marmosets and by an overdose of isoflurane (Pfizer) inhalation for mice. Vascular perfusion was performed using a saline (0.9% NaCl, Sigma, St. Louis, MO, United States) rinse followed by 4% paraformaldehyde (PFA, 16%, Electron Microscopy Sciences, PA, United States), pH 7.4, in phosphate buffered saline (PBS from 10×, Nacalai tesque, Kyoto, Japan) that had been chilled on ice (Step #1 in Table 1). Perfusion with the fixative (300 ml and 30 ml) was conducted at approximately 20 ml/min and 2 ml/min for marmosets and mice, respectively. The target area in the brain tissue was dissected into coronal slices 3–6 mm thick using a 76 μm-thick cutting blade (Nisshin EM Co., Ltd., Tokyo, Japan) and a marmoset brain matrix. The sectioning matrix specific for marmoset brain was designed from three-dimensional (3D) data obtained from magnetic resonance imaging (MRI) (Figure 2A,B). The pieces of the perfused brain were postfixed with 4% PFA in 0.1 M PB, pH 7.4 for 10–12 h at 4°C. Tissue blocks were cryoprotected by incubation in 15% and then 30% sucrose solutions (Nacalai tesque, Kyoto, Japan) for 12 h each and embedded into cryomolds (Tissue-Tek, Sakura Finetek, Tokyo, Japan) with cryocompound (Leica Biosystems, Wetzlar, Germany) for subsequent cryostat sectioning. Frozen sections (20 μm thick in this case) were prepared using a cryostat (Leica CM3050s, Leica Biosystems, Wetzlar, Germany), placed on adhesive microscope slides and dried on a hot plate for 2–3 h at 37°C until the sections were tightly attached to the slides (Step #2 in Table 1 and Figure 2C,D). Folding and wrinkling of the tissue sections inhibit the preparation of ultrathin sections after resin embedding, making it important to ensure that the sections are as flat as possible so as to obtain larger flat samples for EM observation. The dried sections on the slides were stored in either a -30 or a -80°C freezer in a cryosection box until antibody staining on experimental day 1 (Step #3 in Table 1 and Figure 2E). The type of coating on the slides and the materials from which the slides were constructed are critical for LA-CLEM. For general immunostaining, microscope slides have multiple adhesive coatings and are positively charged to keep the section tightly fixed to the slides even after exposure to solution for several days. In some cases and depending on the coating conditions, it may be difficult to remove tissue sections from the slides, as described in Step #36 in Table 1. Prior to experiments involving samples of limited availability, trial removal of the resin should be performed to confirm error-free processing. Glass slides, which are commonly used in immunostaining due to their flatness, hardness, and limited autofluorescence, can also be used in the LA-CLEM procedure. However, it is easier to remove specimens from plastic slides, and these are also sometimes used in immunostaining, as described in Step #36 in Table 1.

**Table 1:** Detailed procedure for LA-CLEM imaging with mSEM.

Step	Prior to starting the experiment	Duration	Temp.	Note
#1	Perfusion of the animal with fixative (4% PFA, etc.)		r.t./4°C	
#2	Preparation of frozen sections (10–20 μm) on glass/plastic slides		-30°C	
#3	Store in freezer		-30/-80°C	
Step	Experimental Day 1	Duration	Temp.	Note
#4	Dry with cool dryer and line drawing for liquid blocking	10–30 min	r.t.	See step #29
#5	Wash 3× with 0.1 M PBS	3 min × 3 min	r.t.	
#6	Pretreatment for antigen retrieval (citrate, TRS in autoclave, MW)	10 min	105°C	
#7	Wash with 0.1 M PBS	3 min	r.t.	
#8	Blocking (5% BlockAce, 0.01% saponin)	30 min ∼ 1 h	r.t.	Described in Figure 2D
#9	**Ms/Rab/Chick/Rat/Goat/Human/Guinea pig 1st Abs**	3 ∼ 4 o/n	4°C	Summarized in Figure 3C
	**application**	(72–96 h)		
Step	Experimental Day 2	Duration	Temp.	Note
#10	Wash 10× with 0.1 M PB and 0.005% saponin	10 min × 10 min	r.t.	
#11#11′	**Gold- and fluorescence-conjugated 2nd Abs application (Category 4 in Figure 3C)Biotin-conjugated 2nd Abs application (Category 5 in Figure 3C)**	1 o/n1 o/n	4°C4°C	Select step #11 or 11′ depending on the host of 1st Ab and on 2nd Ab lineup
Step	Experimental Day 3 (only for Step #11′ Category 5 Abs in Figure 3C)	Duration	Temp.	Note
#12	Wash 10× with 0.1 M PB and 0.005% saponin	10 min × 10 min	r.t.	
#13	**Gold- and fluorescence-conjugated streptavidin**	1 o/n	4°C	Described in Figure 3C
Step	Experimental Day 4	Duration	Temp.	Note
#14	Wash 10× with 0.1 M PB and 0.005% saponin	10 min × 10 min	r.t.	
#15	Fluorescence imaging with light microscope		r.t.	Described in Figure 4A, 7A–C
#16	Wash with 0.1 M PB	5 min	r.t.	
#17	Fix with 2.5% glutaraldehyde	1 h	r.t.	
#18	Wash with 0.1 M PB	5 min	r.t.	
#19	Wash 3× with 50 mM HEPES (pH 5.8)	10 min × 3 min	r.t.	
#20	Silver enhancement with R-gent Se-EM kit (Aurion)	30–40 min	r.t.	Described in Figure 5A–D
#20′	Silver enhancement with HQ-silver kit (Nanoprobes)	10–12 min	r.t.	In the dark room
#21	Wash 5× with DW and 1× with 0.1 M PB	1 min × 6 min	r.t.	
#22	Fix with OsO_4_	1.5–2 h	4°C	
#23	Wash with DW	5 min	4°C	
#24	Dehydration with EtOH (50% ×2)	5 min × 2 min	4°C	
#25	En bloc staining with 2% uranyl acetate (UA) in 50% EtOH	20 min	4°C	
#26	Dehydration with EtOH (70% ×2)	5 min × 2 min	4°C	
#27	Dehydration with EtOH (80% ×2)	5 min × 2 min	4°C	
#28	Dehydration with EtOH (90% ×2)	5 min × 2 min	r.t.	
#29	Dehydration with EtOH (100% ×2) + liquid blocking line removal	5 min × 2 min + α	r.t.	Described in Figure 5E
#30	Acetone	5 min	r.t.	These steps are specific for slide glasses/glass chamber slides/glass vials


#31	QY1 (*n*-butyl-glycidyl-ether)	5 min × 2 min	r.t.	
#32	QY1:Epon = 1:1	1 h	r.t.	
#30′	100% EtOH : 100% Epon = 3:1	10 min	r.t.	These steps are specific for plastic chambers/plastic culture dishes


#31′	100% EtOH : 100% Epon = 1:1	10 min	r.t.	
#32′	100% EtOH : 100% Epon = 1:3	10 min	r.t.	
#33	100% pure Epon	1 h	r.t.	
#34	100% pure Epon	1 o/n	4°C	
Step	Experimental Day 5	Duration	Temp.	
#35	100% pure Epon embedding (with slide-embedding mold)	72 h (3 o/n)	60°C	Described in Figure 5F–H
Step	Experimental Day 6	Duration	Temp.	
#36	Tissue removal from slide glasses on the hot plate		100°C	Described in Figure 6A–D
#37	Block preparation on the sectioning stage	1 o/n	60°C	Described in Figure 6E–G
Step	Experimental Day 7	Duration	Temp.	
#38	Store in desiccator	1–2 h	r.t.	
#39	Block trimming with blade/glass knife/diamond trim knife		r.t.	Described in Figure 6H
#40	Sectioning with ultramicrotome (30–90 nm)		r.t.	
#41	Section collection on tape/silicon wafer/copper grid		r.t.	Described in Figure 6I–K
#42	Dry in desiccator	1–2 h	r.t.	
Step	Experimental Day 8	Duration	Temp.	
#43	Staining with uranyl acetate (UA)	10 min	r.t.	With silicon wafer holder or grid stick


#44	Wash 3× with DW	1 min × 3 min	r.t.	
#45	Staining with lead citrate (Pb)	10 min	r.t.	
#46	Wash 3× with DW	1 min × 3 min	r.t.	
#47	Dry on clean filter paper	1–2 h	r.t.	
Step	Experimental Day 9	Duration	Temp.	
#48	Electron microscopic observation with mSEM/SEM/TEM			Described in Figure 7, 8

*mSEM, multibeam SEM; MW, microwave; Ms, mouse; Rab, rabbit; Ab, antibody; o/n, overnight; r.t., room temperature (24–25°C); 2 h, 2 hours*.

## Immunohistochemical Preparation with Antibody Application

The LA-CLEM procedure was based on pre-embedding iEM, as previously described (Shibata et al., 2015b); the samples were subsequently analyzed by multibeam SEM. On day 1, frozen sections were thawed and dried under a cool dryer for 10–30 min at room temperature (r.t.; Step #4 in Table 1). Before applying blocking solution, we often use liquid blocker or a pap pen to create a barrier that keeps the blocking solution and antibody solution on the section and to prevent contamination of the slides by other solutions or leakage from the slide top. Dried sections were washed with 0.1 M PB 3 times for 3 min at r.t. and then placed in a moist chamber (Step #5 and Figure 2F).

Specific pretreatments for immunostaining, including antigen retrieval using an autoclave and microwaving, can be applied to the sections before the blocking solution is applied (Step #6 in Table 1). Depending on the requirements for each antibody, pretreatment for antigen retrieval was conducted in special solutions, such as pH 6.0 citrate buffer, by heating the slides in a heat-resistant staining pot in an autoclave or microwave. Other commercially available solutions, including pH 6.0 target retrieval solution (TRS from DAKO), should be evaluated on a sample-by-sample basis. It is necessary to allow approximately 1 h for the sections and solution to cool completely to room temperature before performing the PBS wash for 3 min (Step #7 in Table 1).

Blocking solution with or without detergent (saponin, Merck, Darmstadt, Germany) was applied to the sections to block non-specific antibody binding (Step #8 in Table 1 and Figure 2F). We usually used 5% BlockAce (DS Pharma Biomedical, Osaka, Japan) with 0.01% saponin (Merck, Darmstadt, Germany) in 0.1 M PB (Muto Pure Chemicals, Tokyo, Japan) for 0.5–1 h. Commercially available blocking solutions other than BlockAce, such as Blocking Reagent (PerkinElmer, MA, United States) and Blocking Buffer (ab126587, Abcam, Cambridge, United Kingdom), can be used for blocking. The use of donkey, goat, and horse serum is also acceptable if the species from which the primary antibody is derived differs from the species from which the serum used in blocking was derived. It is critical to use a detergent such as saponin, Triton X-100 (FUJIFILM Wako Pure Chemical Corporation, Osaka, Japan), Tween (Sigma, St. Louis, MO, United States), or SDS (Nacalai tesque, Kyoto, Japan) at the minimum required concentration for smooth antibody infiltration. The stronger the detergent we used, the lower was the signal from cell membranes detected with EM due to the breakdown of the lipid bilayer by active permeabilization with detergent.

**Figure 3:**
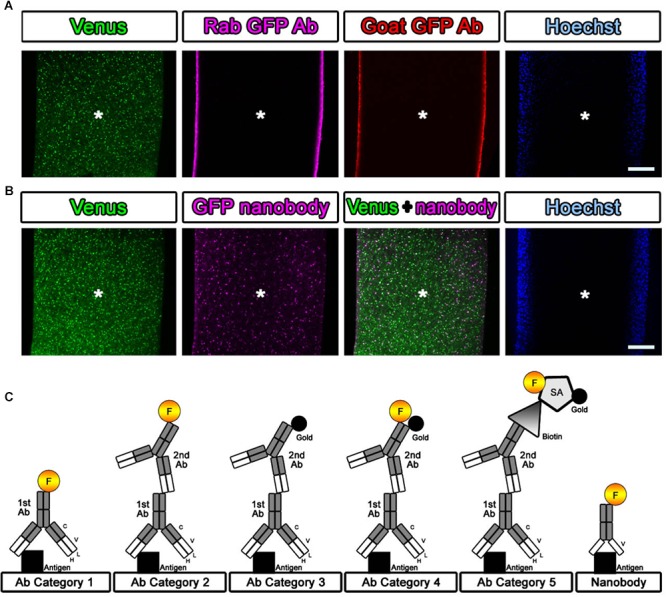
Evaluation of the antibody and nanobody. **(A,B)** Lack of detergent application completely inhibited the infiltration of the antibody. Antibodies did not penetrate into the center area of the tissue, but the nanobodies partially labeled the GFP prepared without detergent at a depth of several hundred micrometers. Asterisk: center of the brain section. Scale bars: 200 μm. **(C)** Categorization of antibodies and nanobodies. Fluorescence labeling with antibody was usually performed in one step using a direct fluorescence-conjugated primary antibody (Category 1) or in two steps using a fluorescence-conjugated secondary antibody (Category 2). For EM observation, gold labeling or DAB (3,3′-diaminobenzidine tetrahydrochloride) reaction with HRP (horseradish peroxidase) was required to visualize the antibody localization (Category 3). In this study, fluorescence- and gold-conjugated secondary antibodies were mainly used (Category 4). When an appropriate dual-labeled secondary antibody was not available, the use of biotin-conjugated secondary antibodies and fluorescence- and gold-conjugated streptavidin helped complete the procedure (Category 5).

If detergent is not used in antibody staining, tissue preservation should be much better for EM observation. However, it is difficult for antibodies to enter brain tissue. We attempted to evaluate the penetration of the antibodies and nanobodies in the absence of detergent and after minimal detergent treatment (Fang et al., 2018). The cerebral cortices of Sox10-Venus transgenic mice (Shibata et al., 2010) were dissected, and coronal brain slices 800 μm thick were prepared promptly after perfusion of the animals with 4% PFA pH 7.4 in PBS on ice using a vibrating blade microtome (Leica VT1000 S, Leica Biosystems, Wetzlar, Germany). After blocking with blocking solution containing no detergent, the slices were stained with antibodies and nanobodies, followed by fluorescence labeling with secondary antibodies and Hoechst dye (Hoechst 33258, Sigma, St. Louis, MO, United States), respectively (Figure 3A,B). Stained brain sections were vertically sliced in the sagittal dimension at 100 μm thickness, and the penetration of the fluorescence was evaluated. Antibodies did not infiltrate sections several micrometers in thickness without detergent, but fluorescence-conjugated nanobodies penetrated brain tissue to a depth of several hundred micrometers (Figure 3B) (Fang et al., 2018). Nanobodies are a promising immuno labeling reagent due to their small size; however, the available selection of nanobodies and gold labeling systems is currently very limited compared to the selection of antibodies, which number more than a million. As described in Steps #9–11 in Table 1 and Figure 3C, the selection of the procedure mainly depended on the purpose of the experiment and the available reagents (antibodies and nanobodies). The main focus of this study is to identify the cerebral cortical layer positions of neurons in large marmoset brain sections using well-known and widely used antibodies.

Sections incubated with blocking solution (5% BlockAce, DS Pharma Biomedical, Osaka, Japan) with 0.01% saponin for permeabilization were incubated with primary antibodies in blocking solution for 3–4 days at 4°C (Step #9 in Table 1, same as Figure 2F). Dilution of the antibodies should be evaluated by light microscopy using a solution similar to that used in LA-CLEM. In this study, the following layer-specific primary antibodies were used: rabbit anti-calbindin (1:500, Chemicon, Merck, Darmstadt, Germany), mouse anti-calretinin (1:200, Swant Inc., CH-1723, Switzerland), chick anti-Tbr1 (1:100, Chemicon, Merck, Darmstadt, Germany), mouse anti-neurofilament H (1:250, clone N52, Sigma, St. Louis, MO, United States), anti-RORβ (1:200, Perseus Proteomics Inc., Tokyo, Japan), rabbit anti-Cux1 (1:500, Santa Cruz Biotechnology, Inc., Dallas, TX, United States), and goat anti-FoxP2 (1:200, Santa Cruz Biotechnology, Inc., Dallas, TX, United States). LA-CLEM can be adapted for use in any host animal (mouse, rabbit, chick, rat, guinea pig, sheep, goat, human, etc.) by using gold- or biotin-conjugated secondary antibodies (Table 2).

**Table 2:** List of the antibodies and nanobodies used in this study.

Primary antibody	Property	Company	Host	Catalog number	Dilution
Anti-calbindin	Neuronal subpopulation cell marker	Chemicon, Darmstadt, Germany	Rabbit Polyclonal	AB1778	1:500
Anti-calretinin	Neuronal subpopulation cell marker	Swant, CH-1723 Marly 1, Switzerland	Mouse Monoclonal	6B3	1:200
Anti-Tbr1, T-box brain protein 1	Neuronal subpopulation transcription marker	Chemicon, Darmstadt, Germany	Chicken Polyclonal	AB2261	1:100
Anti-neurofilament 200 (phosphorylated and non-phosphorylated)	Neuronal cytoskeleton marker	Sigma, St. Louis, MO, United States	Mouse Monoclonal	N0142	1:250
Anti-RORβ, RAR related orphan receptor β	Neuronal subpopulation transcription marker	Perseus Proteomics, Tokyo, Japan	Mouse Monoclonal	N7927-00	1:200
Anti-Cux1, cut-like homeobox 1	Neuronal subpopulation transcription marker	Proteintech, Rosemont, IL, United States	Mouse Monoclonal	11733-1-AP	1:200
Anti-FoxP2, forkhead box protein P2	Neuronal subpopulation transcription marker	Santa Cruz Biotechnology, Dallas, TX, United States	Goat Polyclonal	sc-21069	1:200
Anti-GFP (green fluorescent protein)	GFP, EGFP, and Venus protein labeling	MBL (Medical and Biological Laboratories), Nagoya, Japan	Rabbit Polyclonal	Code 598	1:500
Anti-GFP (green fluorescent protein)	GFP, EGFP, and Venus protein labeling	Rockland, PA, United States	Goat Polyclonal	600-101-215	1:200
Anti-VE-cadherin	Endothelial cell marker	Santa Cruz Biotechnology, Dallas, TX, United States	Goat Polyclonal	(C-19) sc-6458	1:200

**Secondary antibody**	**Property**	**Company**	**Host**	**Catalog number**	**Dilution**

Alexa Fluor 488- and Nanogold-conjugated goat anti-mouse/rabbit IgG	Species-specific IgG detection	Thermo Fisher Scientific, MA, United States	Goat Polyclonal	A25920/A24922	1:100
Alexa Fluor 488- and Nanogold-conjugated streptavidin	Biotin-specific detection with streptavidin	Thermo Fisher Scientific, MA, United States	Streptavidin	A24926	1:100
Biotinylated donkey anti-goat/chicken IgG	Species-specific IgG detection	Jackson Immuno Research, West Grove, PA, United States	Donkey Polyclonal	705-065-147/703-066-155	1:500
Biotinylated goat anti-rat IgG	Species-specific IgG detection	Vector Laboratories, Burlingame, CA, United States	Goat Polyclonal	BA-9400	1:500
Alexa Fluor 555-conjugated donkey anti-rabbit IgG	Species-specific IgG detection	Thermo Fisher Scientific, MA, United States	Donkey Polyclonal	A31572	1:800
Alexa Fluor 647-conjugated donkey anti-goat IgG	Species-specific IgG detection	Thermo Fisher Scientific, MA, United States	Donkey Polyclonal	A21447	1:800

**Nanobody**	**Property**	**Company**	**Host**	**Catalog number**	**Dilution**

GFP-Booster_Atto594 (green fluorescent protein)	GFP, EGFP and Venus protein labeling	ChromoTek, NY, United States	Recombinant	Gba-594-100	1:200

On day 2, we washed the samples 10 times for 10 min each time with 0.1 M PB and 0.005% saponin for a total of approximately 2 h at r.t. (Step #10 in Table 1). While washing, secondary antibodies were prepared for Step #11 by dilution in blocking solution containing BlockAce with 0.01% saponin in PB, as described above. In our laboratory, we used a FluoroNanogold-conjugated secondary antibody (Alexa Fluor 488- and Nanogold-conjugated goat anti-mouse or anti-rabbit antibody, 1:100, Thermo Fisher Scientific, MA, United States) for mouse and rabbit primary antibodies (Step #9 in Tables 1, 2). For detecting antibodies prepared in other species, such as chick, rat, guinea pig, sheep goat, and human antibodies, fluorescence and gold dual-labeled secondary antibodies are commercially available, and biotin-conjugated secondary antibodies (1:500, Jackson Immuno Research, West Grove, PA, United States, or Vector Laboratories, CA, United States) with fluorescence and gold dual-labeled streptavidin can be used (Step #11 in Table 1 and Figure 3C). Alternatively, a 1- or 1.4-nm colloidal gold-conjugated secondary antibody (Nanoprobes Inc., NY, United States) can also be used for Step #11 in Table 1 in conjunction with the fluorescence-conjugated secondary antibodies (Table 2).

In the case of the samples in Step #11 in category 5 in Figure 3C, high-affinity binding of biotin to streptavidin was applied on experimental day 3. After 10 washes with 0.1 M PB and 0.005% saponin, the sections were incubated with FluoroNanogold-conjugated streptavidin (Alexa Fluor 488- and Nanogold-conjugated streptavidin, 1:100, Thermo Fisher Scientific, MA, United States) for 24 h at 4°C (Steps #12 and #13 in Table 1) along with Hoechst 33258 (10 μg/ml, Sigma, St. Louis, MO, United States) for nuclear staining. The inclusion of these additional steps (#12 and #13) on day 3 meant that the total experimental schedule had to be adapted to the host species from which the primary antibodies were obtained and to the lineup of secondary antibodies. In our case, the busiest experimental day, day 4, was usually fixed first, and the schedule for applying primary and secondary antibodies was adjusted later depending on the host species from which the primary antibody was obtained.

## Fluorescence Imaging with Light Microscopy

**Figure 4:**
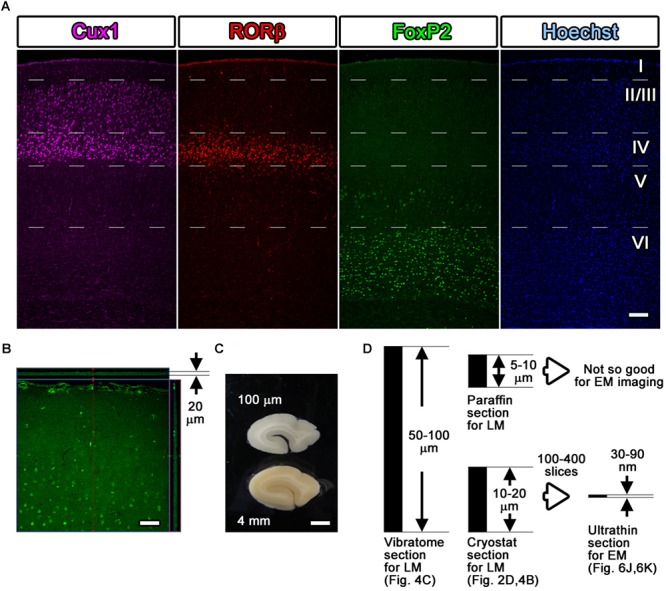
Fluorescence images with layer markers. **(A)** Multicolor fluorescence images were obtained from immunostained mouse brain samples using a confocal laser scanning microscope. Mouse somatosensory cerebral cortex sections were stained with antibodies recognizing Cux1 (magenta, mainly layer II–IV), RORβ (red, mainly layer IV), and FoxP2 (green, mainly layer VI) and with Hoechst dye (blue, nucleus). The white dotted lines indicate the estimated border of each layer. **(B)** An immunostained section was observed by LM, and the thickness and the depth of infiltration by the antibody were evaluated. The full thickness of 20 μm was completely infiltrated with the green-labeled secondary antibody, reflecting the Cux1 localization. **(C)** Floating sections 100 μm thick were prepared from a dissected marmoset brain using a vibratome. The floating sections were frequently used for fluorescence immunostaining of large sections. **(D)** Summary of the thickness of the sections. Vibratome slices and cryostat sections can be transferred for use in CLEM imaging, but the floating sections from the vibratome are too thick to be infiltrated to their full depth. Scale bars: **(A)**–**(B)** 100 μm, **(C)** 2 mm.

After washing several times with 0.1 M PB and 0.005% saponin for approximately 2 h in Step 14, the immunostained samples were observed using a confocal laser scanning microscope (LSM880, Carl Zeiss, Oberkochen, Germany) or a fluorescence microscope (BZ-9000, Keyence, Osaka, Japan) on experimental day 4 (Steps #14 and #15 in Table 1 and Figure 4A, 7A–C). The sections were soaked in buffer (0.1 M PB) rather than in mounting medium. To avoid damage to the sections due to direct contact with the cover glass, we maintained a small space between the slide glass and the cover glass by attaching adhesive tape or positioning an additional cover glass at the edge of the slide glass to create an artificial space. To identify the nuclear localization and tissue structure during fluorescence imaging, nuclear staining dyes (Hoechst or DAPI) were usually included in the secondary antibody solution. Moreover, multicolor imaging with differently colored dyes can be conducted simultaneously by identifying the other epitopes using additional sets of primary and secondary antibodies in Steps #9, #11, and #13.

As an example, four-color fluorescence images were obtained using Hoechst (blue), anti-FoxP2 (green), anti-RORβ (red), and anti-Cux1 (far red), and EM images were obtained using FoxP2 (gold) labeling. Goat anti-FoxP2, mouse anti-RORβ and rabbit anti-Cux1 antibodies were applied as a set of primary antibodies on day 1. Biotin-conjugated donkey anti-goat secondary antibody (1:500, Jackson Immuno Research, West Grove, PA, United States) was applied on day 2. The Hoechst dye, FluoroNanogold-conjugated streptavidin (Alexa Fluor 488 and Nanogold), Alexa Fluor 555-conjugated goat anti-mouse IgG and Alexa Fluor 647-conjugated goat anti-rabbit secondary antibodies were applied on day 3 (Figure 4A).

As demonstrated in Figure 4B, which shows a lateral side view of a section with *Z*-stack imaging after the Cux1 antibody reaction, 20-μm-thick cryostat sections were fully infiltrated by the primary and secondary antibodies without any gaps (Figure 4B). The thickness of cryostat sections varies from laboratory to laboratory. In our laboratory, sections were usually prepared at a thickness of 50–100 μm for free-floating vibratome sections, 10–20 μm for frozen cryostat sections, 5–10 μm for paraffin sections, 50–80 nm for resin-embedded EM sections, and 30–90 nm for resin-embedded SEM observation (Figure 4C,D). Cryostat sections 20 μm in thickness were the maximum thickness that allowed complete infiltration of the antibody described in Steps #9 and #11 in Table 1; this thickness is also ideal for ultrathin sectioning with an ultra-microtome for TEM and SEM observation as described in Step #48 in Table 1.

## Section Processing for EM Block Preparation

Soon after completing the fluorescence imaging, the sections were washed with 0.1 M PB for 5 min at r.t. and fixed with 2.5% glutaraldehyde in PB for 10 min at r.t. for EM-grade fixation (Steps #16 and #17 in Table 1). To minimize exposure to the vapor produced by the toxic reagents and reduce the amount of solution required for each process, a plastic slide case that holds five slides (MR-500, Matsunami glass, Osaka, Japan) is convenient for processing, especially for glutaraldehyde fixation at Steps #16–#18 and for osmium staining, dehydration and Epon infiltration at Steps #22–#34. The use of dummy empty slides to fill the empty wells helps reduce the amount of solution required.

**Figure 5:**
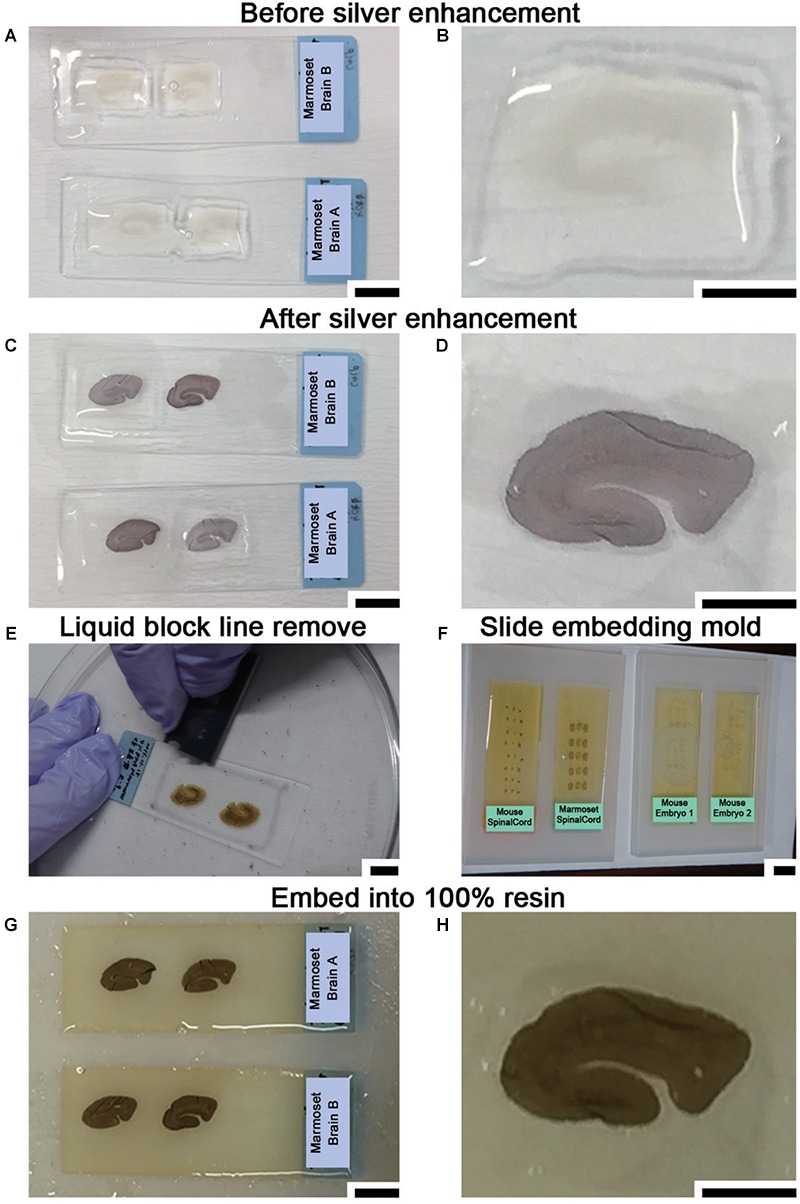
Sample preparation for LA-CLEM using resin blocks. **(A,B)** Silver enhancement was required to visualize the localization of specific molecules using nanogold-conjugated antibody signals. **(C,D)** The tissue became slightly darker when the silver enhancement procedure was completed. **(E)** At the middle of the dehydration step with 100% EtOH, the lines on the slide glass applied with liquid blocker should be removed from the top using a single-edged razor blade. If this step is omitted, it may be difficult to remove the section smoothly from the slide glass after resin polymerization. **(F)** Dehydrated and resin-infiltrated samples were embedded in a silicone mold for slides. Air bubbles under the slides should be removed before beginning polymerization. **(G,H)** Brain slices stained with antibody were fully polymerized by incubating at 60°C for 72 h. Scale bars: **(A,C,E)**–**(G)** 1 cm; **(B,D,H)** 5 mm.

The sections were washed again with 0.1 M PB for 5 min at r.t. and buffered with 50 mM HEPES (FUJIFILM Wako Pure Chemical Corporation, Osaka, Japan) (pH 5.8) for half an hour (10 min × 3 min) at r.t. (Steps #18–#19). Adjustment of the pH for this buffer used 1 N NaOH since Cl^-^ ions from hydrochloride (HCl) generate a white precipitate with Ag^+^ ions that increases the background. Silver enhancement was required to enlarge the Nanogold or 1-nm colloidal gold signal due to the small size of these reagents. Silver enhancement was conducted using an R-gent Se-EM kit (Aurion, PD Wageningen, Netherlands) and developed for approximately 30–40 min at r.t. in a bright room or with the HQ-silver kit (Nanoprobes Inc., Yaphank, NY, United States) for approximately 10–12 min at r.t in a dark room (Step #20 or #20′ in Table 1). When the R-gent Se-EM kit was used, 10 or 20 droplets of activator and one droplet of the initiator (10:1 or 20:1) were mixed well by vortexing to prepare the developer, and 50 droplets of the enhancer and 10 or 15 droplets of developer (10:2 or 10:3) were mixed with vortexing to prepare sufficient reaction solution for processing five slides (Figure 5A–D). When the HQ-silver kit was used in the dark room, 20 droplets of solution A and 20 droplets of solution B were mixed well; 20 droplets of solution C were then added to the tube and mixed well with vortexing to prepare sufficient solution (1:1:1) for processing 5 slides. Stopping of the enhancement reaction was determined by the timing of the color change of the sections to brown or gray. To stop the silver enhancement reaction, the sections in a slide basket were actively washed five times in distilled water (DW) at r.t. for 1 min followed by a wash in 0.1 M PB for 1 min at 4°C in the slide glass plastic case (Step #21 in Table 1). The sections were postfixed with osmium tetroxide (OsO_4_, Nisshin EM Co., Ltd., Tokyo, Japan) for 90–120 min at 4°C, and 15 ml solution was used for 5 slides in a plastic case (Step #22 in Table 1). After removal of the osmium solution, the slide case was washed with DW once for 5 min followed by two incubations with 50% EtOH for 5 min each time (Steps #23 and #24 in Table 1).

To enhance the membrane contrast of the EM images for the whole block, en bloc staining with 2% UA solution in 50% EtOH was performed for 20 min at 4°C (Step #25 in Table 1). As found in various trials to improve EM image quality and summarized in Table 3, en bloc staining with UA was one of the most effective factors in our trial. Dehydration in graded concentrations of ethanol (70, 80, 90, and 100% EtOH) was performed twice at each concentration for 5 min (Steps #26–29). At the step in which the sample is exposed to absolute ethanol (100% EtOH), the lines from the liquid blocker should be removed from the tops of the slides using a razor blade while the slides are immersed in a 10- or 15-cm plastic dish filled with absolute ethanol to facilitate smooth removal of the section after polymerization (Step #29 in Table 1 and Figure 5E).

**Table 3:** Evaluated approaches for LA-CLEM.

Method	Glass/	Pretreatment	Detergent	Post GA	Post OsO_4_	UA	LM	Removal	EM	EM	Overall
	Plastic			fixation	fixation	block	imaging	from	imaging	membrane	LM/EM
	slide					staining		slide	area	contrast	image quality
	
	(Step #2)	(Step #6)	(Step #8)	(Step #17)	(Step #22)	(Step #25)	(Step #15)	(Step #36)	(Step #37)	(Step #48)	Whole steps
(a)	Glass	AC in citrate	0.01% Sap	2.5% GA 1 h	1.3% OsO_4_/Collidin for 2 h	+	Good	Difficult	Narrow	High	High
(b)	Glass	AC in citrate	0.01% Sap	2.5% GA 1 h	1.3% OsO_4_/Collidin for 2 h	-	Good	Difficult	Narrow	Low	Moderate
(c)	Glass	AC in TRS	0.01% Sap	2.5% GA 1 h	1.3% OsO_4_/Collidin for 2 h	+	Very good	Difficult	Narrow	Low	Moderate/Low
(d)	Glass	AC in TRS	0.01% Sap	2.5% GA 1 h	1.3% OsO_4_/Collidin for 2 h	-	Very good	Difficult	Narrow	Low	Low
(e)	Glass	AC in TRS	0.01% Sap	2.5% GA 1 h	Reduced OsO_4_ for 2 h	+	Very good	Very difficult	Narrow	Moderate	Moderate/Low
(f)	Glass	AC in citrate	0.01% Sap	2.5% GA 1 h	Reduced OsO_4_ for 2 h	+	Good	Very difficult	Narrow	Very high	High
(g)	Glass	AC in citrate	0.01% Sap	2.5% GA 1 h	1% OsO_4_/PB for 1.5 h	+	Good	Normal	Large	High	High
(h)	Glass	AC in citrate	0.01% Sap	2.5% GA 20 m	1% OsO_4_/PB for 1.5 h	-	Good	Normal	Large	Moderate	Moderate
(i)	Glass	MW in citrate	0.01% Sap	2.5% GA 1 h	1% OsO_4_/PB for 1.5 h	+	Bad	Normal	Large	Moderate	Low
(j)	Glass	MW in citrate	0.01% Sap	2.5% GA 20 m	1% OsO_4_/PB for 1.5 h	+	Bad	Normal	Large	Moderate	Low
(k)	Glass	MW in citrate	0.01% Sap	2.5% GA 1 h	1% OsO_4_/PB for 1.5 h	-	Bad	Normal	Large	Moderate	Low
(l)	Glass	MW in citrate	0.01% Sap	2.5% GA 20 m	1% OsO_4_/PB for 1.5 h	-	Bad	Normal	Large	Moderate	Low
(m)	Glass	–	0.01% Sap	2.5% GA 1 h	1% OsO_4_/PB for 1.5 h	+	Very bad	Normal	Large	High	Low
(n)	Glass	–	0.01% Sap	2.5% GA 1 h	1% OsO_4_/PB for 1.5 h	-	Very bad	Normal	Large	High	Low
(o)	Glass	AC in citrate	0.1% Sap	2.5% GA 20 m	1% OsO_4_/PB for 1.5 h	-	Good	Normal	Large	Low	Low
(p)	Glass	AC in citrate	0.1% Triton	2.5% GA 20 m	1% OsO_4_/PB for 1.5 h	-	Good	Normal	Large	Very low	Low
(q)	Glass	AC in citrate	–	2.5% GA 20 m	1% OsO_4_/PB for 1.5 h	-	Very bad	Normal	Large	Moderate	Low
(r)	Plastic	AC in citrate	0.01% Sap	2.5% GA 1 h	1% OsO_4_/PB for 1.5 h	-	Good	Quite easy	Very large	Very low	Moderate
(s)	Plastic	AC in TRS	0.01% Sap	2.5% GA 20 m	1% OsO_4_/PB for 1.5 h	-	Very good	Quite easy	Very large	Very low	Moderate

*AC, Autoclave 105 degrees, 10 min; MW, Microwave boil; Sap, Saponin; GA, Glutaraldehyde; Collidin, 2,4,6-Trimethylpyridine; UA, Uranyl acetate; Orange highlight: beneficial effect; Blue highlight: negative effect*.

To replace the solution with 100% Epon for polymerization, acetone was applied for 5 min at r.t. followed by the application of QY1 twice for 5 min at r.t. (Steps #30 and #31 in Table 1). The sections were exposed to the resin-containing solution QY1:Epon (1:1) for 1 h at r.t. and then to 100% pure resin several times at r.t. (Steps #32 and #33) and overnight at 4°C (Step #34 in Table 1). Steps #30 to #32 were usually used only with glass slides, glass chamber slides, and vials made of glass with sufficient solvent resistance. Because plastic slides have limited resistance to solvents, plastic slides should not be exposed to acetone or to QY1. Sections on plastic slides were exposed to gradually increasing concentrations of the resin in absolute ethanol [100% EtOH:100% Epon = 25% (3:1), 50% (1:1), 75% (1:3) for 10 min each at r.t. (Steps #30′–#32′ in Table 1]. The sections were then exposed to 100% Epon for 1 h at r.t. and incubated overnight at 4°C (Step #33). At the final step before beginning the polymerization (Step #34), the slides on which the sections were mounted were transferred to a new plastic slide case containing 100% pure resin to minimize carryover from the previous solvent.

At least one overnight infiltration of 100% pure Epon was used both for slides made of plastic and for slides made of glass; the slides were then embedded in new resin in a slide glass embedding mold made of silicone (SNP2, microstar, Tokyo, Japan) for 72 h (approximately three overnights) at 60°C for polymerization (Step #35 in Table 1 and Figure 5F). To achieve complete curing of the resin (Figure 5G,H), it is important to maintain the optimal temperature for the resin (60°C in our case, from datasheet) for a sufficient period. We monitored the actual temperature on a minute-by-minute basis during the entire period using a temperature probe. When the temperature reached 60°C, approximately 1 h after the slides were placed in the oven for polymerization, the air bubbles under the slides were removed using small wooden toothpicks to maintain the thickness of the resin on the section.

## EM Block Sectioning and Wafer Preparation

**Figure 6:**
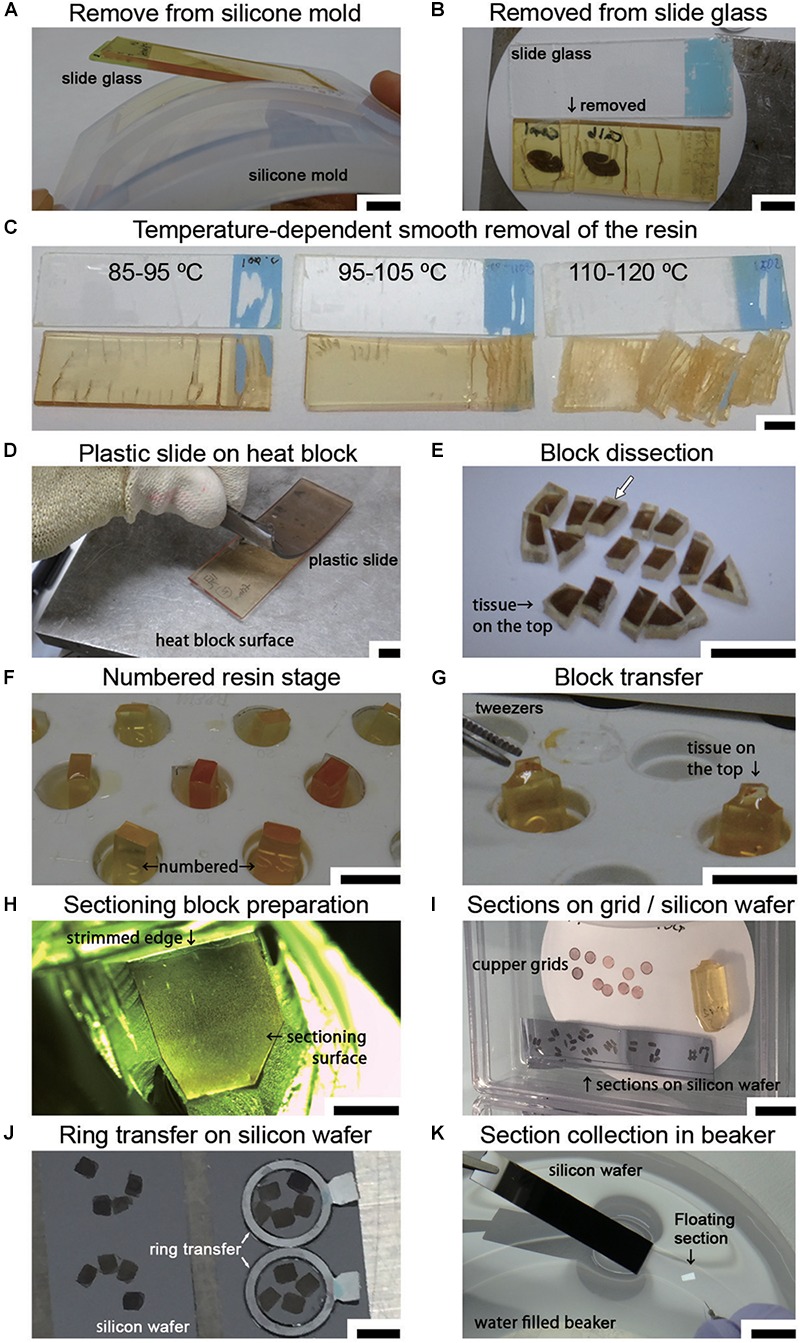
Sample preparation for LA-CLEM on tape or wafers. **(A)** Polymerized resin with slides is easily removed from the silicone slide mold. **(B)** The sections in the resin were removed from the slide glass on a hot plate using a sectioning blade. **(C)** The temperature of the section on the hot plate was critical for the smooth removal of the section from the glass slide. Heating the resin to 90–100°C yielded the best results. **(D)** A plastic slide (plastic chamber slide) softened on the hot plate and was smoothly detachable from the resin. **(E)** Removed sections were dissected on a hot plate into several-millimeter-square blocks using a fine sectioning blade for ultrathin sectioning with a diamond knife. The white arrow shows the block imaged in Figure 7. **(F)** Used resin, which has a high viscosity, was placed on the empty resin block in the capsule stand to serve as a kind of “glue.” **(G)** Tissue sections on the sectioning block were placed on the top of the sectioning stage with small forceps. The glued blocks with the sections on top were incubated for 24 h at 60°C until the sections were strongly attached. **(H)** Blocks were trimmed using a sectioning blade, glass knife, or diamond trimming knife, and ultrathin sections were prepared using an ultra-microtome or an ATUMtome. **(I)** Ultrathin sections were manually collected on copper grids or silicon wafers. **(J,K)** For SEM observation, sections were transferred to a silicon wafer from the diamond knife boat using a ring transfer or by manual collection in a water-filled beaker. Scale bars: **(A–G)** 1 cm, **(H)** 2 mm, **(I)** 1 cm, **(J)** 3 mm, **(K)** 1 cm.

After curing was completed, the sections in the polymerized resin were manually removed from the silicone mold (Figure 6A). Using sectioning blades, the sections were removed from the slides on the hot plate by an experimenter wearing anti-injury gloves that were resistant to cutting (Step #36 in Table 1 and Figure 6B). The temperature of the sections and the coating of the slides were crucial for smooth removal (Figure 6C). The temperature setting of the hot plate should be adjusted depending on the type of resin and the hot plate. With our resin composition, an iron top hot plate (Taitec Corporation heat block, Saitama, Japan) was set to approximately 100°C. This method was well suited to removal of the specimen at 95–105°C as measured by an infrared thermometer. If the resin was not sufficiently hot (85–95°C), it was difficult to remove the specimen from the slide. If it was too hot (>110°C), the resin was easily fragmented into small pieces, presenting the worst condition for block preparation. It is simple to remove resin-embedded specimens from slides made of plastic on a hot plate with limited use of blades (Figure 6D).

Soon after completing the smooth removal and with the sample on the same hot plate, the sections were dissected into blocks several millimeters square (Step #37 in Table 1 and Figure 6E). Using small forceps, the blocks were placed on the empty resin block (sectioning stage) and glued to the block using old resin with high viscosity (stored in the freezer in a syringe, Nipro, Osaka, Japan). The tissue sections should be placed on top of a uniquely numbered sectioning stage (Figure 6E–G; the white arrow in Figure 6E indicates the target section in the experiment at this time point). The block should never be placed upside down. The glued blocks were incubated for 24 h at 60°C. After fixing on the block, the sectioning stage with a piece of the section on the resin block was stored in a desiccator for at least several hours in a paper sample storage box to prevent loss (Step #38).

Ordinarily, one block would be sufficient for large-area imaging; however, we always fix all small blocks on the sectioning stages for two important reasons: to permit numbering of all the small blocks, and to preserve the adjacent blocks in case these are needed. There is unique numbering on the lateral side of the sectioning stage made up of resin blocks (Figure 6F). Both the original position of the brain section and the block number information are always clearly recorded in a notebook. This makes it easier for us to determine the original location of the immunostained section. If we do not place small pieces of blocks on the stage and save them separately in a paper storage box, it is difficult to identify the original position of the antibody-stained section on the resin block. In some cases, it is necessary to prepare additional ultrathin sections from adjacent blocks due to the occurrence of cracks, breaks, or bumpy surfaces that interfere with ultrathin sectioning. For this reason, most of the fluorescence imaged area, at least, is usually prepared as blocks for EM sectioning. The blocks were trimmed to a size of several millimeters square using a sectioning blade, a glass knife or a diamond trimming knife in an ultramicrotome (Leica UC7, Leica Biosystems, Wetzlar, Germany or RMC ATUMtome, Boeckeler Instruments, Inc., Tucson, AZ, United States) for preparing ultrathin sections (Step #40 in Table 1). The size of the sectioning surface was determined by the object, the width of the diamond knife and the flatness of the surface. To obtain full-layer sections from marmoset cerebral cortex, a surface with an area of approximately 2 mm × 3 mm was sufficient to cover all layers (from layers I to VI). To distinguish the pial surface from the ventricular side of the sample, it is useful to prepare the block surface in a trapezoidal shape or in a home base shape that makes it easy to identify the top of the brain.

Ultrathin sections were prepared at a thickness of approximately 30–90 nm using a diamond knife in an ultramicrotome at r.t. (approximately 24°C) (Step #40). Ultrathin sections prepared using the ATUMtome (Boeckeler Instruments, Inc., Tucson, AZ, United States) were collected on conductive tape, manually transferred to silicon wafers or collected on copper grids (Step #41 in Table 1 and Figure 6I). The thickness of the sections was set using the ultramicrotome and was limited by the type and hardness of the resin. For large-area imaging with SEM, ultrathin sections with a thickness of 50–80 nm were prepared, and the sections were transferred to the silicon wafer from the diamond knife boat using a ring transfer (microstar, Tokyo, Japan) (Figure 6J). For section collecting, a silicon wafer or tape for ATUMtome that had been treated with plasma to obtain a clean, hydrophilic surface was used. The larger samples were transferred from the knife boat using a water-filled beaker (Figure 6K). The diamond knife boat containing several sections was dipped into a water-filled beaker. The sections floating on the water were collected directly onto the silicon wafer or conductive tape. For TEM observation, sections approximately 50–80 nm in thickness were manually collected on a copper grid (#100 or #150 Veco, Nisshin EM Co., Ltd., Tokyo, Japan). All sections were dried completely in a desiccator for several hours at r.t. (Step #42).

Ultrathin sections were prepared for EM using UA for 10 min at r.t. and lead citrate (Pb) for 10 min. Sections on a tape of approximately 5–10 cm in length (depending on the staining tube), sections on silicon wafers attached to a silicon wafer holder (microstar, Tokyo, Japan), and sections on copper grids attached to a grid staining stick (microstar, Tokyo, Japan) were dipped into UA solution for an optimal period of time at r.t. (Step #43 in Table 1). The sections were then washed three times with DW in a beaker at r.t. (Step #44). The sections were dipped into Pb solution at r.t., followed by three washes with DW (Steps #45 and #46). The ultrathin sections on the tape and on the silicon wafer were gently blown with an air brush, the remaining water droplets were removed from the top, and the sections were dried completely on clean filter paper for several hours at r.t. (Step #47 in Table 1).

## Electron Microscopic Imaging with Multibeam SEM

**Figure 7:**
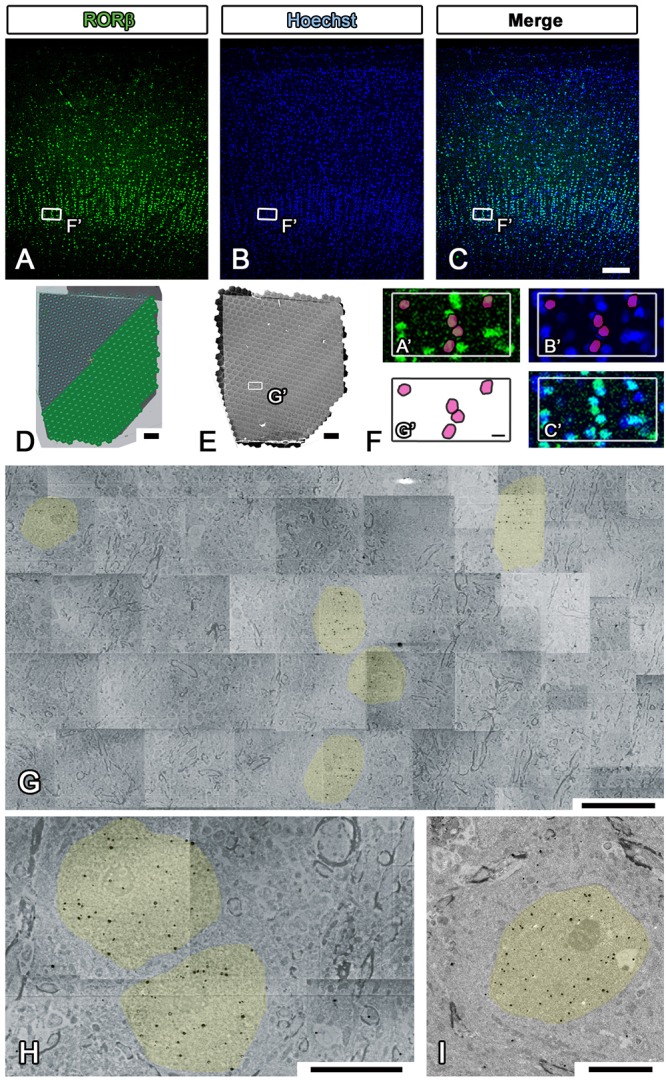
Imaging of whole marmoset cerebral cortex using multibeam SEM. **(A–C)** All fluorescence images shown in the figure were obtained by using a light microscope (LM) to observe the same section of the occipital lobe of the marmoset cerebrum. The small white boxes in each fluorescence image show the position of the enlarged area in **(F)**. **(D)** Our strategy for covering the entire imaging area of the marmoset cerebral cortex with multiple hexagons originated from the 61 split electron beams. **(E)** The whole tiled image was obtained with multibeam SEM from a marmoset brain section labeled with specific brain layer markers. The small white box demonstrates the position of **(G)**. **(F)** A direct overlay of the fluorescence image in **(A–C)** and the EM image in **(G)** by manual correlative observation is shown here. The red circles in **(F)** originate from the yellow-colored area in **(G)** and were superimposed on **(A′)** and **(B′)**. **(G)** Low-magnification EM images of the marmoset brain revealed the position of aggregation of an RORβ-positive layer IV neuron, the nucleus of which is labeled with silver- enhanced gold particles (black dots, colored yellow). **(H)** High-magnification image of the brain section showing the subcellular localization of gold particles (nucleus, yellow). **(I)** Image of an adjacent section on the EM grid acquired with TEM; it exhibited similar localization of gold particles in the nucleus. Scale bars: **(A–C)** 100 μm, **(D,E)** 200 μm, **(F,G)** 10 μm, **(H,I)** 5 μm.

Electron microscopic observation was conducted with multibeam SEM (multiSEM 505, Carl Zeiss, Oberkochen, Germany), single-beam SEM (SU6600 from Hitachi High-tech, Tokyo, Japan, Sigma from Carl Zeiss, Oberkochen, Germany), and TEM (JEM1400plus JEOL, Tokyo, Japan) according to the manufacturer’s instructions (Step #48 in Table 1). For LA-CLEM imaging, multibeam SEM is one of the most powerful microscopic techniques available for use in high-speed imaging of large sections of brain tissue. The sections on the collecting tapes were first placed on the silicon wafer with conductive double-sided adhesive tape. The silicon wafers containing the sections were attached to the specimen holder with silver DAG and imaged using an optical microscope, Imager Vario (Carl Zeiss, Oberkochen, Germany), to confirm the precise position at low magnification as a reference position from which the fluorescence images were obtained (Step #15 in Table 1 and Figure 7A–C). After establishment of the workflow with multibeam SEM so as to observe the brain sections with the best focus and with the best acquisition parameters, the experiment was begun with automatic focus acquisition of a sufficient number of tiled images to cover the target section (Figure 7D). The mosaic image files were automatically generated soon after completing the imaging (Figure 7E). On zooming into the specific region indicated by the white box in Figure 7A–C,E, the localization of the fluorescently stained nucleus was clearly demonstrated (Figure 7F). Low-magnification EM images can be used to identify the specific nuclear localization (yellow) of the RORβ (Figure 7G). In Figure 7F, the red circles in (G′) originate from the yellow-colored nucleus in Figure 7G and are superimposed on the fluorescence images shown in (A′) and (B′). The pattern of the red circles in Figure 7F does not completely match the fluorescence and Hoechst images in Figure 7A,B due to the difference in thickness of the immunostained cryostat sections and ultrathin sections. The higher-magnification imaging demonstrated that the gold signals were mostly localized in the nucleus and enabled the observation of myelin and synapses (Figure 7H). To confirm the detailed structure of the tissue using another microscope at different magnification, single-beam TEM observation of adjacent ultrathin sections placed on the grids was conducted (Figure 7I).

**Figure 8:**
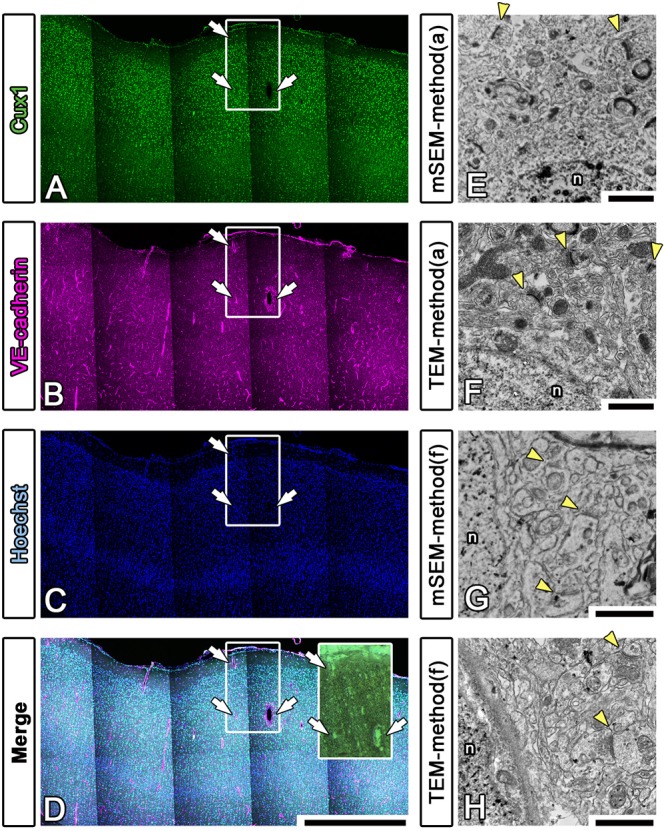
Fluorescence imaging of landmarks and comparison between multibeam SEM and TEM. **(A–D)** Multiply stained fluorescence images were obtained using a confocal laser scanning microscope in tiling mode. The localization of the pia mater (physical edge), blood vessels (VE-cadherin) and nucleus (Hoechst) provided useful clues for finding the precise location of the EM block because these were visible in EM observation. Inset: actual surface image of the resin block corresponding to the white square windows in panels **(A–D)**; the white arrows indicate landmark blood vessels. **(E–H)** As shown in Table 3, various trials were conducted to find methods for improving the quality of the LM and EM images. The EM images shown in **(E,F)** were produced using method (a), and the images shown in **(G,H)** were produced using method (f) in Table 3. The images were evaluated by multibeam SEM **(E,F)** and by TEM **(F,H)**. n, nucleus; arrowheads, synapse. Scale bars: **(A–D)** 1 mm, **(E–H)** 1 μm.

To enhance the accuracy of the overlay between the fluorescence and EM images of large brain sections, we performed multicolor immunostaining using landmark markers, including markers for blood vessels (VE-cadherin, Santa Cruz Biotechnology, Dallas, TX, United States), the nucleus (Hoechst) and the pia mater (physical edge) (Figure 8A–D). As shown in the inset in Figure 8D, the EM block surface also contained a superjacent section of the pia mater and many blood vessels (white arrows) of various diameters. These positional clues are effective not only for our manual correlative observations but also for the computer-based correlative analysis by AI in future.

We also sought to evaluate various approaches that could be used to enhance the image quality of our LA-CLEM observations. A limited number of sample preparation conditions are summarized in Table 3. The overall LM/EM image quality obtained using different conditions, including the use of glass or plastic slides, pretreatment for antigen retrieval, detergent use, postfixation glutaraldehyde and OsO4, and UA en bloc staining, was compared. For example, whereas autoclaving with citrate buffer pH 6.0 is one of the best procedures for antigen retrieval, autoclaving in target retrieval solution (Dako) was powerful and effective for LM imaging but harmful for EM imaging, offering limited microstructure preservation. One of the best procedures tested was method (a) in Table 3. The images shown in Figure 8E,F were obtained with multibeam SEM and TEM, respectively. The application of reduced OsO_4_ in the postfixation step was also effective for drastic enhancement of the membrane contrast (Figure 8G,H); however, the resin-embedded sections were stuck to the slide glass very rigidly and were very difficult to remove. Identifying better conditions for improving the quality of the images for large sample observation using the LA-CLEM procedure remains an important challenge.

## Discussion

In this study, we report a newly developed procedure, LA-CLEM, that can be used to visualize specific molecular localizations in large areas of the CNS at EM resolution and at high speed through the use of multibeam SEM. Information on layer markers in the EM images was helpful for identifying cortical layers in a given region, especially in the cerebrum of the common marmoset. This method may make it possible to rapidly observe large biological specimens, including specimens of human tissue, at EM-level resolution while obtaining information about molecular localization.

Correlative light and electron microscopy has often been used to obtain a correlation between images from LM and EM in very limited areas. In previous reports, various practical approaches using cultured cells or transgenic animal models such as *Drosophila melanogaster*, *Caenorhabditis elegans*, zebrafish, and mouse have been described (Karreman et al., 2016). To identify molecular position at higher resolution, CLEM technology combined with immuno-EM could overcome the limitations of LM by compensation with EM imaging (Cortese et al., 2009), although it is not easy to identify the same area or the same cell using both LM and EM at different magnifications in the same specimen. This is one of the reasons why general CLEM imaging has remained focused on limited areas.

There are two major approaches to obtaining images by EM, SEM, and TEM; these two methods detect signals using scattered electrons and transmitted electrons, respectively. Typically, SEM reveals the surface micromorphology of the specimen, while TEM can be used to visualize the internal composition of thin sections. Due to the basic strategy of the SEM/TEM image acquisition procedures, SEM is more suitable than TEM for the observation of larger areas. TEM can maximally observe an area of up to several mm in diameter within the EM grid at one time, while SEM can scan areas of approximately several cm^2^. Because CLEM imaging has usually been conducted with TEM, the observable area has remained limited (Chen et al., 2012; Kubota et al., 2015).

In contrast, TEM delivers much higher resolution than SEM; however, SEM technology is gradually improving and is now approaching the resolution of TEM. Recent advances have made it possible to obtain images of the internal composition of thin sections on a flat surface that are quite similar to those obtained using TEM (Marx, 2013). In this study, we sought to observe large areas of ultrathin sections of marmoset and mouse brain with SEM by detecting the secondary electrons from the surface of the sample. To increase throughput for numbers of large sections from the brain, we used multibeam SEM, increasing the number of primary beams and detectors to enlarge the imaging area compared to single-beam SEM (Marx, 2013; Eberle et al., 2015b). Multibeam SEM has opened a new era of EM observation, enabling nanoscale resolution imaging of areas on the order of mm^2^ or cm^2^ in size (Marx, 2013). In addition to parallel imaging with a multidetector in a single image, multiple scanning with precise tiling can be used to image the entire surface of large samples (Eberle et al., 2015a). The multibeam SEM that was used in this study achieved extraordinarily high-speed imaging with parallel electron beams. The specifications sheet of the multibeam SEM stated that a 1 cm^2^ area can be imaged within an hour at 4 nm/pixel resolution.

The LA-CLEM procedure introduced in this paper is a novel approach in which CLEM is combined with multibeam SEM. Primary observation with LM was conducted to visualize the fluorescence localization, followed by observation of the same specimen with multibeam SEM at different magnification and resolution. Due to the use of antibody-specific fluorescent labeling, the molecular identity of each labeled cell in the monkey brain can be clearly categorized. Previously, 3D molecular localization in the primate brain was visualized by immunostaining; however, that study was conducted only at the LM level (Mikula et al., 2009). Gold-labeled signals can be detected simultaneously with the detailed intracellular structure revealed by EM, directly confirming the subcellular localization of targets in the nucleus, cytoplasm, cell membrane and synapse at EM resolution. By combining CLEM imaging with multibeam SEM technology in the brain it is possible to identify the localization of specific neuronal subtype markers at the EM level in a large cerebral section, and such localization is important for efficiently determining the function and connection of specific neuron types. It would be highly beneficial to identify layer-specific markers that can provide information at the EM level for evaluating layer-specific connections in the brain.

Classically, SEM provides 3D images, while TEM provides two-dimensional (2D) images. Recent technological advances have made it possible to visualize the 3D structure of a specimen at EM resolution using SEM; the results not only show the irregularity and roughness of the surface but also provide multiple serial imaging of the 2D flat surface (Shibata et al., 2015a; Karreman et al., 2016). Some serial section EM (ssEM) approaches, including focused ion beam (FIB)-SEM, serial blockface electron microscopy (SBEM), and automated tape-collecting ultramicrotome (ATUM)-SEM, are available. The advantages of ssEM with FIB-SEM are that the highest Z resolution available by ultrathin slicing with the FIB is several nm and that the technique is applicable to hard tissues (teeth and bone) that are not suitable for cutting with a diamond knife. Instead of using a FIB, SBEM serial sectioning is performed by using a diamond knife to slice the top surface of the tissue, and the newly created surface is imaged with SEM. In contrast to the destructive techniques of FIB-SEM and SBEM, which destroy the sample as it is being imaged, in ATUM-SEM serial sections are produced by a standard ultramicrotome, collected automatically on tape, and imaged by SEM, offering the possibility of reimaging the same section multiple times if necessary (Kasthuri et al., 2015; Morgan et al., 2016; Hildebrand et al., 2017). In addition, it is possible to observe a larger area with sufficient conductivity by ATUM-SEM using the on-tape conductivity escape from the charge-up. By reconstructing the 3D structure of the sample from the images obtained with multibeam SEM, the LA-CLEM approach will enhance throughput and may become an important tool in the near future.

Whole brain-wide connectomics reconstructed at EM resolution requires novel procedures complemented by precisely evaluated fixation and staining procedures for preserving the cellular ultrastructure throughout the brain and sophisticated data processing protocols for the management of petabyte-scale data (Lichtman and Denk, 2011; Mikula and Denk, 2015; Mikula, 2016; Hildebrand et al., 2017). For the reliable reconstruction of neural circuits, the identification of synapses and the detection of cell bodies are critical. X-ray microcomputed tomography (X-ray microCT) and X-ray microscopy rely on the detection of X-rays transmitted through samples to visualize the internal morphological composition of the block (Bushong et al., 2015; Mikula and Denk, 2015). Improvements in X-ray 3D imaging will help enhance microscale imaging of the whole brain by supplying the information necessary for assessing brain integrity, including the formation of blood vessels and large bundles of nerve tracts (Mikula, 2016).

## Ethics Statement

Housing of animals and all animal experiments were conducted following the Guidelines for the Care and Use of Laboratory Animals of Keio University School of Medicine (approved number 11006-2 and 09091-12), and of the Central Institute for Experimental Animals (approved number 16023 and 17031). All efforts were made to reduce the number of animals used and animal suffering.

## Author Contributions

SS, ES, TT, RS, JL, and HO conceived and designed the analysis. SS, TarI, TM, TS, NM, TN, and TakI conducted the experiments. SS, TarI, TM, TS, NM, TN, TakI, ES, and CA contributed to animal sample preparation. SS, TS, NM, TN, RS, and JL planned and conducted the electron microscopic imaging. SS, TarI, TM, AO, TT, and HO contributed to the interpretation of the results. SS, TarI, TM, AO, SO, and HO wrote the manuscript. All authors provided critical feedback and helped shape the research, analysis and manuscript. All authors approved the final submission of this manuscript.

## Conflict of Interest Statement

The authors declare that the research was conducted in the absence of any commercial or financial relationships that could be construed as a potential conflict of interest.

## Abbreviations

CLEM: correlative light and electron microscopy
CNS: central nervous system
EM: electron microscopy
GFP: green fluorescent protein
iEM: immuno-electron microscopy
ISH: *in situ* hybridization
LA-CLEM: large-area CLEM
LM: light microscope
PB: phosphate buffer
PBS: phosphate buffered saline
r.t.: at room temperature (24–25°C)
SEM: scanning electron microscopy
TEM: transmission electron microscopy
UA: uranyl acetate.
